# Performance Analysis of Extracted Rule-Base Multivariable Type-2 Self-Organizing Fuzzy Logic Controller Applied to Anesthesia

**DOI:** 10.1155/2014/379090

**Published:** 2014-12-21

**Authors:** Yan-Xin Liu, Faiyaz Doctor, Shou-Zen Fan, Jiann-Shing Shieh

**Affiliations:** ^1^Department of Mechanical Engineering and Innovation Center for Big Data and Digital Convergence, Yuan Ze University, Chungli 320, Taiwan; ^2^Department of Computing, Faculty of Engineering and Computing, Coventry University, Priory Street, Coventry CV1 5FB, UK; ^3^Department of Anesthesiology, National Taiwan University Hospital, Taipei 100, Taiwan; ^4^Center for Dynamical Biomarkers and Translational Medicine, National Central University, Chung-Li 32001, Taiwan

## Abstract

We compare type-1 and type-2 self-organizing fuzzy logic controller (SOFLC) using expert initialized and pretrained extracted rule-bases applied to automatic control of anaesthesia during surgery. We perform experimental simulations using a nonfixed patient model and signal noise to account for environmental and patient drug interaction uncertainties. The simulations evaluate the performance of the SOFLCs in their ability to control anesthetic delivery rates for maintaining desired physiological set points for muscle relaxation and blood pressure during a multistage surgical procedure. The performances of the SOFLCs are evaluated by measuring the steady state errors and control stabilities which indicate the accuracy and precision of control task. Two sets of comparisons based on using expert derived and extracted rule-bases are implemented as Wilcoxon signed-rank tests. Results indicate that type-2 SOFLCs outperform type-1 SOFLC while handling the various sources of uncertainties. SOFLCs using the extracted rules are also shown to outperform those using expert derived rules in terms of improved control stability.

## 1. Introduction

Anesthesia is a branch of medical science involved in the administration of anesthetic agents whose aim is to keep patients in a state of insensitivity during surgical procedures. Modern balanced general anesthesia includes muscle relaxation, unconsciousness (i.e., depth of anesthesia), and analgesia (blocking response to pain). The first two are regulated by the anesthetist in the operating theater, while the third is related to postoperative conditions [1–4]. In the past two decades, there have been several studies on applying intelligent systems to regulate and control anesthetic delivery [5–14]. The human body is a highly nonlinear and multivariable system with many sources of uncertainty that make designing such an automatic controller challenging, specifically:

- physiological differences in age, gender, and preoperative health conditions from one person to another (interpatient variability) can all have an effect on the concentration and duration of anesthetic drug that is required to be administered during surgery [1];
- differences in the anesthetic drug concentration required to be infused due to variability in the physiological effects of drugs on the body (pharmacodynamics) and variability in the drugs metabolism in the body (pharmacokinetics);
- dynamic multivariable changes and interactions in the patient's physiological parameters such as heart rate, respiration, blood pressure (BP), and muscle relaxation (EMG) need to be monitored and controlled by the anesthetist during surgery (intrapatient variability);
- noise and variability in signals are sensed and monitored from the human body such as data collected from EMG and BP monitors.

The above sources of uncertainties translate into a high degree of nonlinearity; complex input output relationships; and encountered uncertainties within the control process. Fuzzy logic controllers (FLC) provide a methodology for designing robust controllers that are able to deliver a satisfactory performance while contending with the uncertainty and imprecision attributed to the real world [15, 16]. FLCs transform numerical information into linguistic values and infer output control responses by using fuzzy rules that encapsulate nonlinear relationships between the system inputs and controlled outputs without the need for any mathematical model. FLCs are therefore able to exhibit robustness with regard to noise and variation of system parameters in complex highly nonlinear problem domains such as biomedical control systems [17, 18]. There have been a number of previous applications of FLCs for automated drug infusion control as described in [10, 19, 20]. These systems have used FLCs to control the infusion rates of different drugs based on approximating the outputs of a reference model in a closed loop design. Previous works [6] on applying FLC in anesthesia have mainly use type-1 fuzzy sets, whose grades of membership are crisp and therefore unable to fully handle the uncertainties affecting parameter variability associated with biomedical control processes and in particular controlling anesthesia delivery during surgical procedures. In order to solve the drawbacks of type-1 systems, type-2 fuzzy systems which use type-2 fuzzy sets have been applied to control anesthesia [2]. Type-2 FLCs have the potential to outperform type-1 FLCs and have been shown under specific conditions to produce more accurate and stable control performances in face of different sources of uncertainties [21–24].

Due to the dynamic changes caused by external stimuli's and the effect of different drugs on patients during surgical operations, the fuzzy logic controller also has to adapt its control rules to facilitate regulation and adjustment of administered anesthetic in response to physiological indicators such as BP and level of paralysis to maintain depth of anesthesia (DoA). This is especially important during multistage operation procedures where the DoA is not always kept at the same level and the maintained set points for parameters such as muscle relaxation and BP are changed during surgery. The self-organizing fuzzy logic controller (SOFLC) proposed by Shieh et al. and Procyk and Mamdani [6, 25] is a successful approach that uses a learning algorithm which can generate and modify rules based on the performance of the control system and is well suited to deal with multivariable adaptive control of drug delivery during surgical operations [6]. In an SOFLC, the initial rule-base is an important factor for determining its control behavior and performance. Traditional methods to obtain fuzzy rules have been through consultation with experts (e.g., doctors) [8, 26]. In recent years, there have been some studies on extracting fuzzy rules using machine learning approaches such as genetic algorithm, neural network, and from initial pretraining an SOFLC to determine the most frequently used control rules [27–31]. Extracted rules by SOFLC are proved to have better control performance than the original expert rules under a single variable environment [29]. In [30], Liu et al. extracted a multivariable rule-base for anesthesia control; however, its performance was not fully verified.

In this paper, we propose the use of type-2 SOFLCs for the automatic control of anesthesia during multistage surgical procedures, where the type-2 fuzzy sets are constructed using data acquired from real patients during surgical procedures. We perform unique simulated experiments under signal noise and model uncertainties in which we evaluate the ability of the type-2 SOFLCs in controlling anesthetic drug delivery to maintain physiological set points for muscle relaxation and BP (used in assessing consciousness) based on a nonfixed multivariable patient model for regulating intravenous administration of atracurium and inhaled isoflurane. The control performance of the type-2 SOFLC is evaluated by comparing the pretrained extracted rule-bases based on analyzing rule usage, with the expert designed rule-bases for a simulated multistage surgical procedure. The experiments show how our type-2 SOFLCs produce a better control performance in the face of uncertainties compared to the type-1 SOFLC. The type-2 SOFLCs with the pretrained extracted rules also produce smoother control behavior than that using the expert derived rules.

The rest of paper is organized as follows. In Section 2, we describe the patient anesthetic model used in our simulations. In Section 3, we present the structure and theory of type-2 SOFLC. In Section 4, we present our experiments and results. Finally, the conclusions are given in Section 5.

## 2. Patient Anesthetic Model

Clinically, anesthetists measure the patient's level of sensation based on muscle relaxation measured from electromyogram (EMG) signals. To assess unconsciousness, anesthetists generally use the signal of BP as a reliable source to define the anesthesia level that relates to the DoA [32, 33]. In this paper, in order to maintain these two physiological signals, we use two common drugs, atracurium for controlling muscle relaxation and isoflurane for controlling BP, which follows previous studies [2, 6, 30].

In practice, anesthetists use a pharmacological model to describe and understand the drug's metabolic effects [24]. Modern pharmacological modeling consists of two categories: pharmacokinetics (PK) and pharmacodynamics (PD). The former describes the concentration of drugs in tissue as a function of time and dose schedule, whereas the latter describes the relationship between drugs concentration in blood and its effect [34]. The pharmacological models of atracurium and isoflurane are described as follows.

### 2.1. The Atracurium Mathematical Model

According to previous studies [33, 34], the atracurium pharmacokinetics can be expressed by the following transfer function (1) which describes the pharmacokinetics of the muscle relaxation relating to atracurium:

(1) $$\begin{array}{c} G_{1}\left(s\right)=\frac{9.94\left(1+10.64s\right)}{\left(1+3.08s\right)\left(1+34.42s\right)}. \end{array}$$

The drug's pharmacodynamics effect can be expressed as the following transfer function [35]:

(2) $$\begin{array}{c} G_{11}\left(s\right)=\frac{K_{1}\left(1+T_{4}s\right)e^{-\tau_{1}s}}{\left(1+T_{1}s\right)\left(1+T_{2}s\right)\left(1+T_{3}s\right)}, \end{array}$$

where *τ*_1_ is a dead time (time elapsed until the drug takes effect), *K*_1_ is a coefficient, and *T*_1_, *T*_2_, *T*_3_, and *T*_4_ are time constants with the values: *τ*_1_ = 1 min, *K*_1_ = 1, *T*_1_ = 4.81 min, *T*_2_ = 34.42 min, *T*_3_ = 3.08 min, and *T*_4_ = 10.64 min. In addition, the following Hill equation is used to relate the effect of a specific drug concentration as described in (3) [36, 37]:

(3) $$\begin{array}{c} E_{\text{eff}}=E_{\max}\frac{X_{E}^{\alpha }}{{X_{E}^{\alpha }+\left(X_{E}\left(50\right)\right)}^{\alpha }}, \end{array}$$

where *X*_*E*_ is the drug concentration, *α* is the power, and *X*_*E*_(50) is the drug concentration at 50% effect with the following values: *E*_max⁡_ = 100%, *X*_*E*_(50) = 0.404 *μ*g/mL, and *α* = 2.98.

### 2.2. The Isoflurane Unconsciousness Model

Up till now there is still no direct method to measure DoA since the brain activity is too complicated to observe. Clinically, BP is one of the signs that are commonly used to indicate DoA. Based on previous studies in [6, 38], the responses of BP to inhaled isoflurane concentration is approximately linear when the changes in isoflurane concentration are less than 5%. However, the responses are in general nonlinear and time varying if the changes become large. Therefore, a first-order linear model with a dead time of 0.42 minutes and a time constant of 2 minutes is used. In addition, in order to estimate the steady-state gain, it is assumed that a relatively sensitive patient needs 2% isoflurane for a 30 mmHg reduction in mean arterial pressure. Therefore, the model describing variations of BP to inhaled isoflurane concentration can be written as follows [6]:

(4) $$\begin{array}{c} G_{22}\left(s\right)=\frac{\Delta \text{MAP}\left(s\right)}{U_{2}\left(s\right)}=\frac{K_{2}e^{-\tau_{2}s}}{1+T_{5}s}, \end{array}$$

where MAP is mean arterial pressure, *τ*_2_ is a dead time, *T*_5_ is a time constant, and *K*_2_ is a coefficient with the following values: *τ*_2_ = 0.42 min, *T*_5_ = 2 min, and *K*_2_ = −15 mmHg/percent.

### 2.3. The Interactive Component Model

According to previous studies, the interaction of atracurium to BP is so small that can be ignored [26, 33]. The interaction of isoflurane to muscle relaxation is significant and is expressed by the following equation [39]:

(5) $$\begin{array}{c} G_{12}\left(s\right)=\frac{K_{4}e^{-\tau_{4}s}}{\left(1+T_{6}s\right)\left(1+T_{7}s\right)}, \end{array}$$

where *τ*_4_ is dead time, *T*_6_ and *T*_7_ are time constants, and *K*_4_ is a coefficient having the values: *τ*_4_ = 1 min, *T*_6_ = 2.83 min, *T*_7_ = 1.25 min, and *K*_4_ = 0.27.

### 2.4. The Multivariable Anesthetic Model

Based on (1)–(5) described in previous sections, the overall multivariable anesthetic model combining muscle relaxation (based on the pharmacokinetics and nonlinear pharmacodynamics of atracurium) and unconsciousness (based on the effects of isoflurane on BP) can be summarized as follows:

(6) $$\begin{array}{c} \left[\begin{array}{c} \text{Paralysis}\\ \Delta \text{MAP} \end{array}\right]=\left[\begin{array}{cc} G_{11}\left(s\right) & G_{12}\left(s\right)\\ 0 & G_{22}\left(s\right) \end{array}\right]\left[\begin{array}{c} U_{1}\\ U_{2} \end{array}\right], \end{array}$$

where *U*_1_ is the atracurium infusion and *U*_2_ is the isoflurane concentration.

### 2.5. Nonfixed Anesthetic Model

The traditional fixed patient mathematical model is based on clinical data [33, 34] and cannot represent the dynamic changes of the patient during surgical operations (intrapatient uncertainties) and the difference from one person to another (interpatient uncertainties). Following on from our previous study [30], we added 1% white noise where this value was obtained by trial and error and consultation with experts to approximate the maximum value of possible parametric uncertainty affecting all parameters in (1) to (5) used in our multivariable anesthetic model. By using this nonfixed patient anesthetic model we can account for the possible patient drug interaction uncertainties during our simulations and more suitably test the features of type-2 SOFLCs, in their ability to handle these encountered uncertainties.

## 3. Type-2 SOFLC

**Figure 1:**
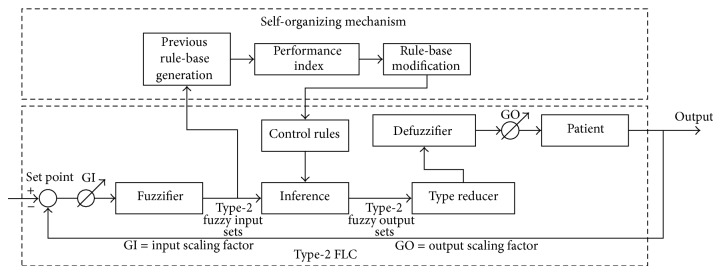
Schematic diagram of a type-2 SOFLC structure.

A type-2 SOFLC has a closed loop hierarchical adaptation and control structure which consists of a type-2 fuzzy logic controller (FLC) based on type-2 fuzzy sets and a self-organizing (SO) mechanism as shown in Figure 1. Each of these components will now be described in the following sections.

### 3.1. Type-2 Fuzzy Sets

**Figure 2:**
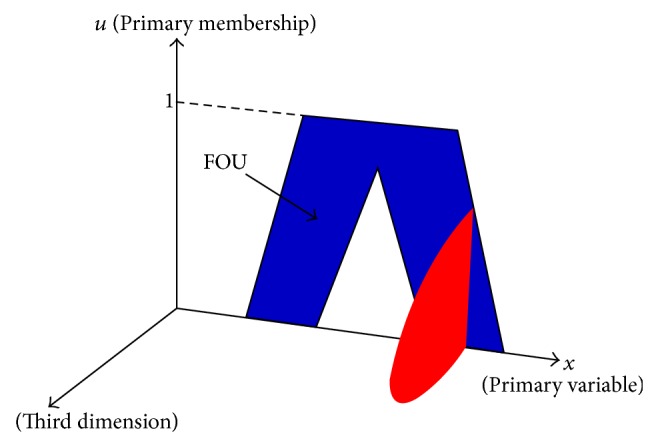
An example of an MF for a general type-2 fuzzy set showing the intersection of an input variable *x* over the FOU where the primary membership values are associated with an amplitude distribution projected in the third dimension forming a secondary MF.

The concept of a type-2 fuzzy set is an extension of type-1 fuzzy set. Unlike a type-1 fuzzy set whose membership grades (or membership values) are a crisp number in [0,1], a type-2 fuzzy set is characterized by a fuzzy membership function (MF), where the membership values for each element of the set are themselves a fuzzy set in [0,1]. Hence for a given input variable *x* to the set, the MF takes on values wherever the vertical line projected for *x* intersects a bounded area known as the footprint of uncertainty (FOU) of a type-2 fuzzy set; see Figure 2. The membership of the type-2 set at *x* therefore comprises the primary membership values that intersect the FOU. Each primary membership value can have a weight associated with it creating an amplitude distribution projected in the third dimension. This distribution forms what is termed as a secondary MF (shown in red) which provides an additional design degree of freedom for modeling higher level uncertainties associated with the primary membership values. Type-2 fuzzy sets are therefore useful in simulating uncertain multivariable systems such as anesthesia control where it is difficult to determine the exact MF for the fuzzy sets due to inter- and intrapatient, pharmacodynamic, and pharmacokinetic variability in the effects of the drugs on the patients bodies. Type-2 FLCs are also able to realize more complex nonlinear input-output control relationships than a type-1 FLC [40, 41] which can be suitable for nonlinear biomedical control processes such as anesthesia regulation.

Currently most practical implementations of type-2 fuzzy sets have been based on using interval type-2 sets [42–44] due to their implementation simplicity where the third dimensional secondary MF is modeled as a fixed interval (interval type-2) as opposed to a continuous fuzzy set (general type-2), whose support is in the interval [0,1] [45]. Recently a new extension to interval type-2 sets called zSlices based general type-2 fuzzy sets [46] has allowed the three dimensional properties of type-2 sets to be more fully realized for practical real world applications. zSlice general type-2 fuzzy sets [46] are formed by slicing a general type-2 fuzzy set into a finite number of interval type-2 fuzzy sets. Thus, the calculations associated with using general type-2 fuzzy set are simplified to those of interval type-2 fuzzy sets [47]. In this paper, type-1, interval type-2, and zSlice general type-2 SOFLC are compared, in terms of their ability to control anesthesia delivery and maintain physiological set points for muscle relaxation and BP while handling the environmental and patient uncertainties during multistage operational procedure.

The FOUs of the SOFLC's input type-2 fuzzy sets are generated using data acquired from monitoring physiological parameters of real anesthetized patients, in order to account for the uncertain parameter variability during DoA control. Average percentage of muscle relaxation and standard deviations and the average BP and standard deviations were collected from 15 anesthetized patients while undergoing ear, nose, and throat (ENT) surgical procedures [7]. The standard deviation from the average of these values sampled for a given patient represents the intrapatient uncertainties over the duration of the surgical procedure. A heuristic process was then applied to generate the FOUs for the interval type-2 fuzzy sets for the type-2 SOFLC parameters. In the case of the zSlices based general type-2 fuzzy sets, this process was extended to then identify similar patients from the data and group them into five groups based on similar values for muscle relaxation and BP. These groups were used to construct five sliced FOUs for representing the uneven interpatient uncertainties over the third dimension to build the zSlices based general type-2 fuzzy sets. The SOFLCs output type-2 fuzzy sets were constructed based on determining the best drug infusion and concentration range to induce patients into anesthesia and then calculating the uncertainty ranges for creating the type-2 FOUs.

### 3.2. Type-2 Fuzzy Logic Controller

The type-2 FLC consisted of a fuzzifier, inference engine, rule-base, type reducer, and defuzzifier as shown in Figure 1 [48]. The input signals from the patient anesthetic model to the controller are taken at each sampling instant in the form of four inputs: the error of muscle relaxation, integration error of muscle relaxation, error of BP, and integration error of BP, which is the same as that in our previous study [30]. The fuzzier transforms crisp data into type-2 fuzzy sets. The inference engine uses rules activated according to input type-2 fuzzy sets to infer the output type-2 fuzzy sets. The type reducer then combines the output type-2 fuzzy sets to form a type-1 fuzzy set which is known as the type reduced set [49]. The defuzzifier is the same as that used in traditional type-1 FLCs, which can defuzzify the type-reduced sets to produce the crisp control outputs [47]. There are two output control signals corresponding to the change of atracurium infusion rate and the change of isoflurane concentration which are based on the integration of these output values in order to facilitate real-time adjustment of anesthetic dosage. The crisp outputs are sent to the patient anesthetic model whose responses are then fed back to the type-2 SOFLC and compared with the set points to calculate the error and integration error of the input control signals.

### 3.3. Self-Organizing Mechanism

The SO mechanism has the ability to generate and modify the control rules to output the desired control responses [6]. It includes three functional blocks: the previous rule-base generation, performance index, and rule-base modification as shown in Figure 1. The previous rule-base generation block uses an initial rule-base at the first sample point that is generated from human expert experience (i.e., anesthesiologist knowledge). During the control process, the rule-base in the previous rule-base generation block will be modified by the SO mechanism itself. The performance index measures the deviation from the desired response and calculates the appropriate changes that are required in the output of the controller. The generation and modification of the control rules are achieved by assigning a credit or reward value (i.e., performance index) to the individual rule combinations defined in a multidimensional performance index matrix, part of which is shown in Table 1 [6, 50]. Rule combinations that will contribute to an improved performance will be added as new rules to the Type-2 FLC rule-base in order to modify the output of the controller in the next control step. The new generated rules at each control step are compared with the existing rules. If the rule is already present, it is ignored. However, if it is a new rule, it will be added into the rule-base. The linguistic performance rules shown partially in Table 1 are based on a qualitative “feel” for the patient and are intended to provide fast convergence around the equilibrium state to achieve a high accuracy. For this reason, they are not specific to the type of patient being controlled and may be similarly defined for different patients. Since it is difficult to handle performance index and control rule-base under multidimensional space, almost all studies of multivariable SOFLC use a method for decomposing an *n*-input/*m*-output system into a set of 2-input/1-output systems [6, 30, 51]. In our simulations, the 4-input/2-output system is also therefore decomposed into more interpretable 2-input/1-output systems. Further details on the SO mechanism can be found in [6, 50].

### 3.4. Rule-Base

The fuzzy rules form part of the type-2 FLC and are manipulated by the SO mechanism as described in Section 2.3. The rule-base contains a set of fuzzy rules used in the fuzzy inference process to infer output control response. In the 2-input/1-output system used in our simulations, the fuzzy rules can be represented as

(7) $$\begin{array}{c} \text{IF}\;\;x\;\;\text{is}\;\;\tilde{F},\;\;y\;\;\text{is}\;\;\tilde{F},\;\;\text{THEN}\;\;z\;\;\text{is}\;\;\tilde{G}, \end{array}$$

where *x* and *y* are inputs, $\tilde{F}$ are the input fuzzy sets, *z* is output, and $\tilde{G}$ is the output fuzzy set. The input fuzzy sets correspond to a series of linguistic labels: negative big (NB), negative middle (NM), negative small (NS), zero (ZE), positive small (PS), positive middle (PM), and positive big (PB), over the ranges of the input variables. The output fuzzy sets correspond to the labels: zero (ZE), positive small (PS), positive middle (PM), and positive big (PB) over the two the output variable ranges.

The SOFLC rule-bases encapsulate the control behavior for regulating anesthesia and our experiments will test the performance of two differently derived rule-bases. One is the traditional rule-base derived from expert experience and is shown in Table 2. The other is an extracted rule-base based on pretraining the SOFLC as described in our previous study [30]. The extracted rule-base is extracted from the SOFLC based on the rule usage analysis and is shown in Table 3. The rule usage analysis analyses the rules based on their firing percentage to decide whether a rule is important or not during the control process. Comparison of the two rule-bases in Tables 2 and 3 shows that the extracted rule-base has a reduced number of rules for describing extreme input conditions such as NB and PB for the error of muscle relaxation and BP; however, there are more rules generated for describing middle level input conditions like NS, ZE, and PS. Such a distribution will have the ability to give more precise control nearer to the set points to be maintained and avoid accidental large or variable dosage injections caused by noise or other operational interference.

## 4. Experiments and Results

We present unique simulations in which we evaluate the type-1, interval and zSlices based general type-2 SOFLC using both the expert derived and pretrained extracted rule-bases. The simulations compared the performance of each controller in their abilities to effectively control the infusion and concentration rates for atracurium and isoflurane to regulate set points for muscle relaxation and BP in the face of noisy signals. A nonfixed multivariable anesthetic model of the pharmacokinetic and pharmacodynamics effects of these drugs, accounting for possible uncertainty variability in these effects, was used as the patient reference model as described in Section 2.

### 4.1. Simulation Methods

Clinically, anesthetists are usually required to apply a different level of DoA and muscle relaxation at different stages of a surgical operation, which is especially true for complex procedures such as neurosurgical and spinal procedures. These kinds of surgeries normally require the patient to be under deep anesthesia during more invasive preliminary stages, while requiring them to be in a shallower anesthetized state when verifying cortical and nervous stimulation. Hence, the surgical anesthesia simulations were designed to run for a duration of 300 minutes (simulating a five-hour procedure) divided into two stages in order to evaluate the controllers' ability to adaptively control the infusion and concentration rates for atracurium and isoflurane to handle set point changes of muscle relaxation and BP.

The set points to be maintained for muscle relaxation were set at 0.8 and 0.9 normalized units at the two stages, respectively, and the set points for BP were set at 100 and 90 mmHg at the two stages, respectively. From the point of view of clinical measurements, in order to measure muscle relaxation, we can place stimulating electrodes for a Datex Relaxograph over the ulnar nerve of the noninfused hand, while sensing electrodes placed over the hypothenar area. The ulnar nerve is stimulated supramaximally with repeated Train-Of-Four (TOF) via surface electrodes at intervals of 0.5 seconds (2 Hz). The TOF stimulus is repeated every 10 seconds to produce the expected degree of neuromuscular block [52]. In our previous work, the initial default values of EMG set points used were 10% and 20% of the baseline for stages 1 and 2, respectively, based on different surgical needs for adequate muscle relaxation [8]. Hence, the same values were used for our simulation of muscle relaxation which corresponded to normalized output values of 0.9 and 0.8, respectively. For measuring BP, previous studies used an MP60 critical care patient monitor to measure patients' MAP at one-minute interval [53]. Hence, mmHg was used for representing BP in our simulations. Normally, MAP during anesthesia will reduce 10~15% in comparison with conscious state of patients. Hence, we simulated the set points of MAP at 100 and 90 mmHg in this study.

In this work, the SOFLC outputs control responses that simulate anesthesia infusion and inhalational rates of atracurium and isoflurane, respectively. However, for controlling infused drugs such as atracurium or cisatracurium, normal clinical practice has been to use a syringe pump, like an Ohmeda 9000 or Graseby 3500, via a computer to control the infusion rate [8]. With inhalational drugs such as isoflurane or desflurane, a stepping motor can be used via a computer to control the inhalational gas concentration [32]. The simulations were run using MATLAB on a laptop with an Intel(R) Core(R) i5-2450M Processor, running MS Windows 7. Each simulation was run for 30,000 intervals where 100 intervals represented 1 minute of time.

In modern surgical procedures, it is usual practice to administer an initial bolus of anesthetic to patients in order to raise its concentration in blood to an effective level in order to reach rapid anesthesia and unconsciousness [54]. In our simulations, an initial bolus is modeled based on atracurium, which is administered to patients to reach the saturation of muscle relaxation. The amount of atracurium injected to the patients is about 5 normalized units for the first 5 minutes. During 5 to 15 minutes, the muscle relaxation which reaches almost saturation settles down to be near to the desired set point for 10 minutes. Because atracurium affects muscle relaxation and has virtually no effect on consciousness (i.e., BP), the patient's BP is maintained constant at 120 mmHg for the first 15 minutes via only the effect of isoflurane. In clinical operations, it is usual to give intravenous administration of propofol as an initial bolus to quickly bring patient into an unconscious state and then use isoflurane to maintain the anesthesia level. However, in these simulations we do not include a model of the effects of propofol on BP, which we consider to be a possibility for future work. Following the initial bolus effect, the SOFLC controller is turned on to control multivariable anesthesia system.

The strength of physiological signals like muscle relaxation and BP is so small that it is susceptible to interference when measuring. In most cases, the amplitude of noise is up to 20% of standard deviation of the signal strength in measuring instruments [55]. In our simulations, we added 10% and 20% white noise to the measured signals (i.e., muscle relaxation and BP values), in order to test the robustness of SOFLCs under real environmental uncertainty.

### 4.2. Comparative Methods

In order to compare the performance of different rule-bases and different types of SOFLC, we measured steady state errors for muscle relaxation and BP (absolute error calculated from the difference between the actual and set point values to be maintained, based on the average values over the last 50 minutes of simulation) and control stabilities (standard deviation of atracurium injection and isoflurane concentration calculated over the entire simulation duration). Since we added white noise to affect physiological signal measurements, which has a random influence on the results, we repeated each simulation 10 times to account for these effects in our analysis. We applied Kruskal-Wallis test to find whether there is difference between type-1, interval type-2, and zSlice general type-2 SOFLCs. In addition, in order to rank the three SOFLCs and compare the expert derived rule-base with the extracted rule-base, we applied Wilcoxon signed rank test. The Kruskal-Wallis test is a nonparametric test that can test whether the mean values of the different groups of data being tested are equal, whereas the Wilcoxon signed-rank test is used for the comparison of two paired samples [56, 57]. In our simulations, we set the significance level *α* = 0.05. Our testing hypothesis for evaluating the SOFLCs was based on the notion that the zSlice general type-2 SOFLC would rank as performing better than interval type-2 SOFLC followed by the type-1 SOFLC according to their uncertainty modeling capabilities. Similarly, we hypothesized that SOFLCs using the extracted rule-bases would perform better than those using expert derived rule-bases. Hence, we applied one-tailed tests to compare these SOFLCs.

### 4.3. Comparison of Type-1, Interval and zSlice General Type-2 SOFLCs

Although the data results of each of the repeated simulations were different due to the added white noise, the simulated drug induced muscle relaxation and BP values of each SOFLC converged to similar values among the 10 repeated simulations. Therefore, we chose typical values corresponding to the 10% and 20% noise that was added to each controller from the 10 simulations to construct our simulation plots for analysis.

**Figure 3:**
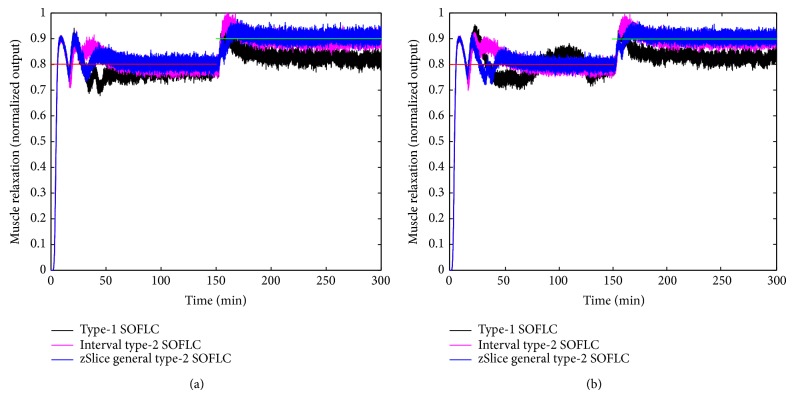
The simulation result of muscle relaxation under 10% noise: (a) expert derived rule-base; (b) extracted rule-base.

**Figure 4:**
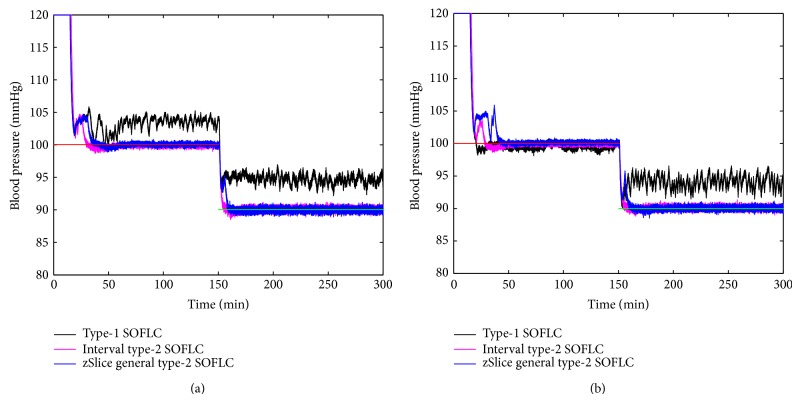
The simulation result of BP under 10% noise: (a) expert derived rule-base; (b) extracted rule-base.

**Figure 5:**
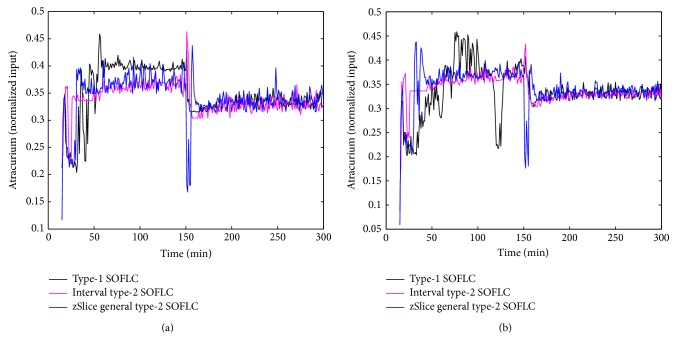
The simulation result of atracurium infusion under 10% noise: (a) expert derived rule-base; (b) extracted rule-base.

**Figure 6:**
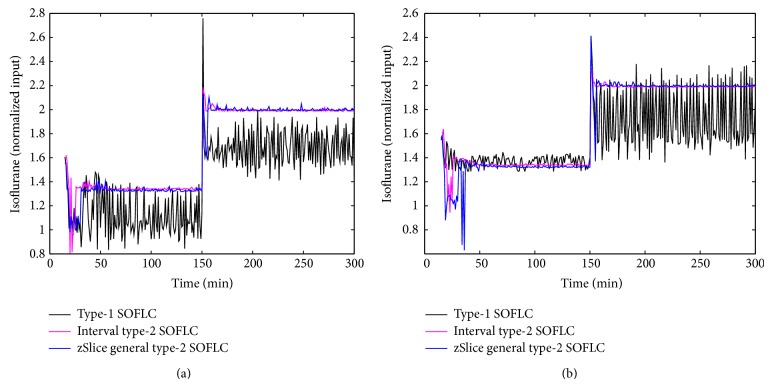
The simulation result of isoflurane concentration under 10% noise: (a) expert derived rule-base; (b) extracted rule-base.

**Figure 7:**
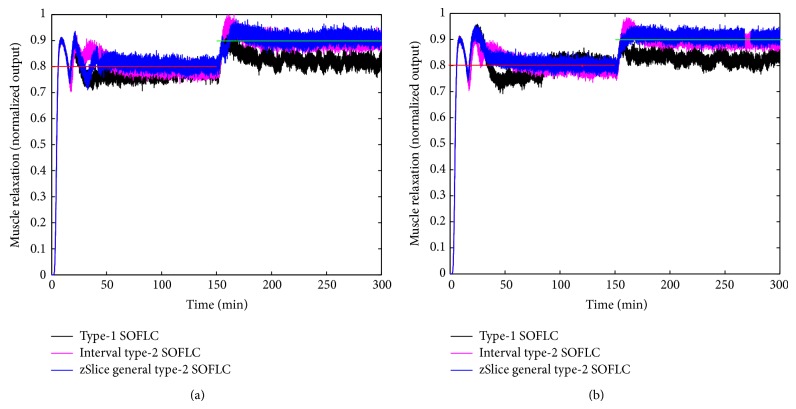
The simulation result of muscle relaxation under 20% noise: (a) expert derived rule-base; (b) extracted rule-base.

**Figure 8:**
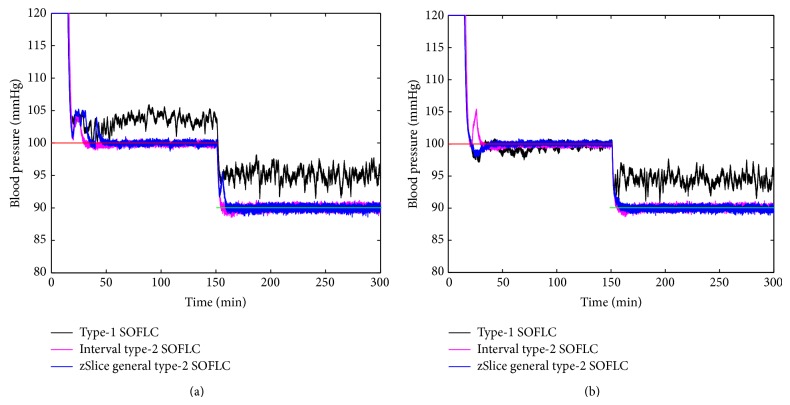
The simulation result of BP under 20% noise: (a) expert derived rule-base; (b) extracted rule-base.

**Figure 9:**
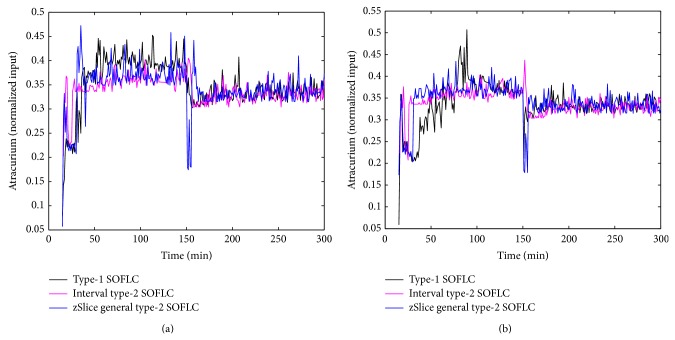
The simulation result of atracurium infusion under 20% noise: (a) expert derived rule-base; (b) extracted rule-base.

**Figure 10:**
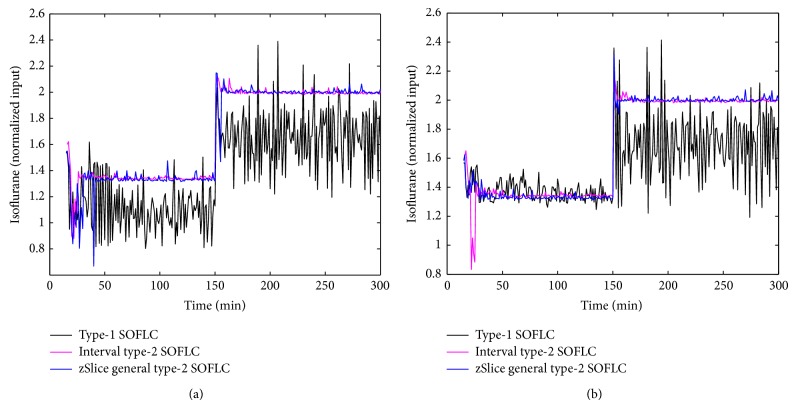
The simulation result of isoflurane concentration under 20% noise: (a) expert derived rule-base; (b) extracted rule-base.

Figures 3(a), 4(a), 5(a), and 6(a) show simulation results of SOFLCs using expert derived rule-base under 10% noise in terms of muscle relaxation, BP, atracurium infusion, and isoflurane concentration, respectively, where the achieved steady state errors and control stabilities are shown in Table 4. In Figure 3(a), we can see that, for type-1 SOFLC, muscle relaxation is close to the desired set point at stage-1, but it has an offset at stage-2, while interval and zSlice general type-2 SOFLC remain steady at the desired set points over both of the two stages. In Figure 4(a), the two type-2 SOFLCs perform better compared to the type-1 SOFLC since they can converge at set points for BP, whereas type-1 SOFLC has an obvious offset. In addition, we can see that in Figure 6(a) the isoflurane concentration of type-1 SOFLC fluctuates much more wildly than type-2 SOFLCs, which is dangerous during a surgical procedure. When we take a look at Figures 7(a), 8(a), 9(a), and 10(a) showing the results for 20% noise cases in terms of muscle relaxation, BP, atracurium infusion, and isoflurane concentration, respectively, the type-1 SOFLC still produces larger control errors than type-2 SOFLCs and also has a significant oscillation in isoflurane concentration. When comparing interval and zSlice general type-2 SOFLC, the results show that the two type-2 SOFLCs produce a comparable steady state control performance.

Table 4 shows the steady state errors and controls stabilities of each SOFLC controller in maintaining two different set points for muscle relaxation and BP while operating under 10% and 20% signal noise. We ran 16 test cases, where each case evaluated the comparative performance difference for each of the three SOFLCs. By applying Kruskal-Wallis test, we found that there existed differences in all the 16 test cases. Then we used Wilcoxon signed-rank test to do pairwise comparison of the three controllers for all the 16 test cases. Three groups of one-tailed Wilcoxon signed-rank tests were applied to test the following hypotheses: (a), (b), and (c) which were interval type-2 being better than type-1, zSlice general type-2 being better than type-1, and zSlice general type-2 being better than interval type-2, respectively; see footnotes below Table 4. Based on the result shown in Table 4, we can see that, when compared with the type-1 SOFLC, both the two type-2 SOFLCs outperform type-1 SOFLC and hence these results accept the first two hypotheses (i.e., interval type-2 is better than type-1 and zSlice general type-2 is better than type-1) in all the 16 test cases. However, when comparing interval and zSlice general type-2 SOFLCs, only 3 of the 16 tests that were carried out gave results supporting the hypothesis, while the other 13 tests rejected the hypotheses. Therefore, these results show that under these specific simulation conditions there was no significant difference between interval and zSlice general type-2 SOFLCs.

### 4.4. Comparison of Expert Derived and Extracted Rule-Base

In order to compare the performances of expert derived and extracted rule-bases, we conducted a second set of simulation studies on the different SOFLCs. The SOFLCs were initialized with the reduced extracted rule-bases derived from the previous simulations in Section 4.3 based on rule usage analysis. Part (b) of Figures 3, 4, 5, 6, 7, 8, 9, and 10 show simulation results of SOFLCs using extracted rule-base under the different added noise values. Comparing (a) and (b) of Figures 3, 4, 7, and 8, we find that the BP offsets of the type-1 SOFLC at stage-1 under both 10% and 20% noise are eliminated. However, other control reactions of muscle relaxation and BP of the extracted rule-bases are comparable to those of the expert derived rule-bases. Comparing (a) and (b) of Figures 5, 6, 9, and 10, the plots of atracurium injection and isoflurane concentration of the extracted rule-base seem to have less fluctuation than the expert derived rule-base.

**Figure 11:**
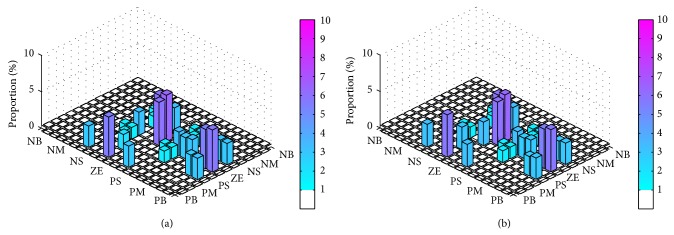
Firing percentage of expert derived rule-base running in zSlice general type-2 SOFLC: (a) atracurium rule-base; (b) isoflurane rule-base.

**Figure 12:**
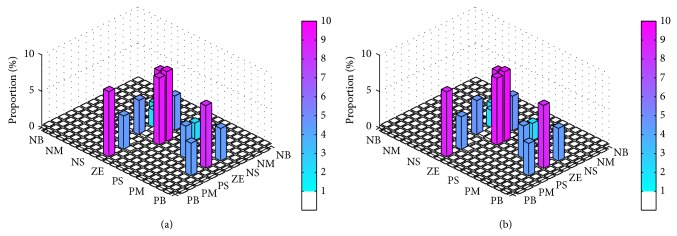
Firing percentage of extracted rule-base running in zSlice general type-2 SOFLC: (a) atracurium rule-base; (b) isoflurane rule-base.

In order to make a precise analysis, we again applied a one-tailed Wilcoxon signed-rank test on the hypothesis that SOFLCs using the extracted rule-base would perform better than those using the expert derived rule-base. We therefore had 24 different test cases based on considering the steady state errors and 24 different test cases based on considering control stabilities of each rule-base (expert derived or extracted) specific controller in maintaining two different set points for muscle relaxation and BP while operating under 10% and 20% signal noise. Note that the 48 test cases (24 for steady state errors and 24 for control stabilities) used for the expert derived rule-base are shown in Table 4 and were used in the previous comparative study of the different SOFLCs described in Section 4.3. The results of the test in Table 5 show that, in terms of steady state errors, 14 of the 24 test cases rejected this hypothesis, which does not give a conclusive result. However, when considering control stabilities, it was found that only 3 of the 24 test results rejected the hypothesis and in all these cases a type-1 SOFLC was used. These results imply that the extracted rule-base can give a smoother control performance which may also be further enhanced by using type-2 fuzzy sets. Following our previous study [30], we recorded firing percentage of rule usage (calculated as the number of times in which a rule was fired divided by total number of inference operations) so as to analyze the importance and contribution of each rule. The firing percentage of the expert derived and extracted rule-bases for the zSlice general type-2 SOFLC running under 20% noise shown in Figures 11 and 12, respectively. The *x*-*y* plane of Figures 11(a) and 11(b) correspond to (a) and (b) of Table 2, respectively, and the height of each bar shows the firing percentage of corresponding rules. Similarly, Figure 12 corresponds to Table 3. The first column on the left and top row in Tables 2 and 3 represent the linguistic labels for the error and integration error, respectively, of each input parameter (muscle relaxation and BP). The intersection of each identical set of these linguistic labels forms a block of six cells which represent the output linguistic label corresponding to the six decomposed 2-input combinations (i.e., error of muscle relaxation and integration error of muscle relaxation, error of muscle relaxation and error of BP, error of muscle relaxation and integration error of BP, integration error of muscle relaxation and error of BP, integration error of muscle relaxation and integration error of BP, and error of BP and integration error of BP), as shown in Tables 2 and 3. Each of the cells in Tables 2 and 3 therefore represents a decomposed 2-input/1-output rule whose firing percentage is shown in Figures 11 and 12, respectively, as described above. Since those rules that were fired less than 1% of the total number of inference operations were mainly fired due to noise, we considered them to be trivial rules and set them to be white in Figures 11 and 12. By comparing Figures 11 and 12, we can see that the rules in Figure 12 have a deeper color than those in Figure 11, which indicates that the extracted rule-base rules contribute more frequently to the inference operations than expert derived rule-base. The complete list of rules' firing percentage of the expert derived rule-base and extracted rule-base (corresponding to each of the decomposed 2 input 1 output systems) can be found in Tables 6 and 7, respectively, in which trivial rules are not included so as to save space.

## 5. Conclusions

In this paper, we have proposed a system for automatically controlling anesthesia during multistage surgical procedures based on type-2 SOFLCs. SOFLCs provide a qualitative adaptive control mechanism for adjusting the controller's behavior through the adaptation of initial expert derived rules that are trained to approximate a desired control behavior. In this case, the type-2 SOFLC simulated control of anesthetic drug delivery to maintain physiological set points for muscle relaxation and BP based on a nonfixed multivariable patient model for regulating intravenous administration atracurium and inhaled isoflurane, in the presence of signal noise. Type-2 fuzzy sets derived from variable patient data were used for modeling the system parameters as they have been credited with producing more accurate and stable control performances in face of different sources of uncertainties [21–24].

The performance of type-1, interval type-2, and zSlice type-2 SOFLC using both the expert derived and pretrained extracted rule-bases for controlling anaesthesia was evaluated based on steady state errors and control stabilities during the simulations. Since we used the nonfixed patient model and added environment noise to our simulations, we repeated each simulation 10 times and applied Wilcoxon signed-rank test to make a precise comparison of their performances. Two groups of comparison were carried out on the different SOFLCs, the first using the expert derived rules and the second using rules extracted from a pretrained SOFLC based on analyzing rule usage and eliminating unused rules. In the first group, the results showed that both the two type-2 SOFLCs produced better performances than type-1 SOFLC. There was however no significant difference between interval and zSlice general type-2 SOFLC in steady state errors and control stabilities, but zSlice general type-2 SOFLC spent less time reaching the set points. In the second group, the SOFLCs with extracted rule-bases produced a smoother control than those with expert derived rule-bases, but from the aspect of steady state error, their performance superiority was not conclusive enough.

Theoretical and practical evidence suggests that zSlice general type-2 fuzzy sets should produce a better control performance than interval type-2 fuzzy sets in situations of high uncertainty paired with a drastic change in inputs and the requirement for a responsive performance [58]. zSlice general type-2 fuzzy sets provide a more accurate model of the uncertainty in the third dimension that should allow for more responsive control while maintaining a smooth control response. However, the results show that zSlices based general type-2 SOFLC does not produce a significant performance difference in simulations compared to using an interval type-2 SOFLC. We consider that this phenomenon is due to following two reasons. Firstly, the performance of interval type-2 SOFLC is already very good indicating that in the context of these simulations there may not be the need to model significantly higher levels of uncertainty using zSlices based type-2 fuzzy sets. Secondly the resolution of the zSlice general type-2 fuzzy sets in terms of the number of slices being used was quite course. In this paper, the zSlice general type-2 fuzzy sets we used were sliced into 5 slices based on the interpatient variability that was encountered from the patient data used to construct them. As suggested in [58], zSlices can be used to model these complex general type-2 sets and associated levels of uncertainty to an arbitrary degree of accuracy that is dependent on the numbers of zSlices [58]. Hence, more accumulated patient data would allow us to construct less granular zSlices based general type-2 fuzzy sets containing a higher number of zSlices as well as more representative shaped FOUs corresponding to the true uncertainty distribution in the data. Future studies may consider whether having more or an optimum number of zSlices will produce a better control performance in the face of higher levels of uncertainty.

The performance difference between the expert derived and extracted SOFLC rule-bases was mainly evident in control stabilities rather than the steady state errors. The extracted rule-base was shown to produce better precision in controlling anesthesia, which produced better control stabilities especially in combination with using the type-2 SOFLCs. This is because at the very beginning of the simulations the extracted rule-base can reach the set point over a steady state more quickly and stably than the expert derived rule-base. However, the similar performance of the two rule-bases in terms of steady state errors can be explained due to the characteristic of the SOFLC to modify the rule-base before reaching a steady state control. However, the extracted rule-base is still able to use fewer rules to reach the same kind of steady state error performance as the expert derived rule-base. This can be an important advantage in terms of reduced real-time computational processing overheads and future deployment of the system on embedded hardware microcontrollers which may have limited memory capabilities. During our experiments, we also recorded rules' firing percentage and the result showed that the extracted rule-base used a subset of fewer more concentrated rules that contributed more frequently and consistently over the inference operations, resulting in better control stability than the expert derived rule-base. In our simulations, we did not use the rules' firing percentage values as additional inputs into our SO mechanism. Further research can use these values to assign a weight to each rule in order to improve the rule modification process. During the simulations we occasionally found that the type-1 SOFLC using the extracted rule-base was less stable compared to using the expert derived rules. As the extracted rule-base has a reduced number of rules, it would indicate that the type-1 SOFLC is not well suited for operating with a highly reduced rule-base. This is due to type-1 fuzzy sets not being able to encapsulate the parametric uncertainty about the membership function as can be achieved using type-2 fuzzy sets due to their FOUs. Therefore, upon the occurrence of extreme conditions, the type-1 SOFLC has to spend time learning how to deal with the condition by generating new rules, whereas type-2 fuzzy sets can continue to operate in these boundary situations even though there are no rules defined for them.

Although we constructed a verisimilar simulation environment, which includes a nonfixed patient model and signal noise, there are still many unknown factors making real surgical environments different from simulations. Presently the type-2 SOFLC can provide a framework for preoperative simulation of anesthesia regulation based on specific surgical conditions and pharmacokinetic and pharmacodynamics effects of the drugs on patient specific physiological characteristics. This can help the anesthetists' preplan dosage and duration variability during surgery. Further research will apply type-2 SOFLCs in clinical settings to evaluate their real world performance.

## Figures and Tables

**Table 1:** SOFLC performance index matrix [6, 50].

Error of muscle relaxation or blood pressure	Integration error of muscle relaxation or blood pressure						
	NB	NM	NS	ZE	PS	PM	PB
NB	NB	NB	NB	NM	NM	NS	ZE
NM	NB	NB	NM	NM	NS	ZE	NS
NS	NB	NB	NS	NS	ZE	PS	PM
ZE	NB	NM	ZE	ZE	PS	PM	PB
PS	NM	NS	ZE	PS	PS	PB	PB
PM	NS	ZE	PS	PM	PM	PB	PB
PB	ZE	PS	PM	PM	PB	PB	PB

Note: PB: Positive big; PM: Positive middle; PS: Positive small; ZE: Zero; NS: Negative small; NM: Negative middle; NB: Negative big.

**Table:** (a) Atracurium rule-base

ATR	NB			NM			NS			ZE			PS			PM			PB		
NB	PB	PB	PB				PS	PM	PS				ZE	ZE	ZE				ZE	ZE	ZE
	PB	PB	ZE				PM	PS	ZE				ZE	ZE	ZE				ZE	ZE	ZE

NM				PM	PM	PM				ZE	ZE	ZE				ZE	ZE	ZE			
				PM	PM	ZE				ZE	ZE	ZE				ZE	ZE	ZE			

NS	PB	PM	PB				PS	PS	PS				ZE	ZE	ZE				ZE	ZE	ZE
	PB	PB	ZE				PM	PS	ZE				ZE	ZE	ZE				ZE	ZE	ZE

ZE				PM	PM	PM				ZE	ZE	ZE				ZE	ZE	ZE			
				PM	PM	ZE				ZE	ZE	ZE				ZE	ZE	ZE			

PS	PM	PS	PB				ZE	PS	PS				ZE	ZE	ZE				ZE	ZE	ZE
	PB	PM	ZE				PS	ZE	ZE				ZE	ZE	ZE				ZE	ZE	ZE

PM				PM	PS	PM				ZE	ZE	ZE				ZE	ZE	ZE			
				PS	PM	ZE				ZE	ZE	ZE				ZE	ZE	ZE			

PB	PM	PS	PB				ZE	PS	PS				ZE	ZE	ZE				ZE	ZE	ZE
	PB	PM	ZE				PS	ZE	ZE				ZE	ZE	ZE				ZE	ZE	ZE

**Table:** (b) Isoflurane rule-base [30]

ISO	NB			NM			NS			ZE			PS			PM			PB		
NB	ZE	PB	ZE				ZE	PM	ZE				ZE	ZE	ZE				ZE	ZE	ZE
	ZE	ZE	ZE				ZE	ZE	ZE				ZE	ZE	ZE				ZE	ZE	PS

NM				ZE	PM	ZE				ZE	ZE	ZE				ZE	ZE	ZE			
				ZE	ZE	ZE				ZE	ZE	ZE				ZE	ZE	PS			

NS	ZE	PS	ZE				ZE	PS	ZE				ZE	ZE	ZE				ZE	ZE	ZE
	ZE	ZE	ZE				ZE	ZE	ZE				ZE	ZE	PS				ZE	ZE	PM

ZE				ZE	ZE	ZE				ZE	ZE	ZE				ZE	ZE	ZE			
				ZE	ZE	ZE				ZE	ZE	ZE				ZE	ZE	PM			

PS	PM	ZE	PM				PS	ZE	PS				PS	ZE	ZE				ZE	ZE	ZE
	PM	PM	ZE				PS	PS	PS				PS	PS	PS				ZE	ZE	PB

PM				PM	ZE	PM				PS	ZE	ZE				ZE	ZE	ZE			
				PM	PM	PS				PS	PS	PM				ZE	ZE	PB			

PB	PB	ZE	PB				PM	ZE	PM				PS	ZE	ZE				PS	ZE	ZE
	PB	PB	PS				PM	PM	PM				PS	PS	PB				PS	PS	PB

**Table:** (a) Atracurium rule-base

ATR	NB			NM			NS			ZE			PS			PM			PB		
NB																					
																				

NM																					
																				

NS							PS	PS	PS	PS	PS			ZE					ZE	ZE	
							PM	PS	ZE				ZE						ZE		

ZE										ZE	PS	ZE				ZE	ZE				
							PS	PS	ZE	PS	PS	ZE				ZE			ZE		

PS																					
							PS	ZE		PS											

PM																					
							PS			PS	ZE	ZE									

PB																					
							PS	ZE		ZE											

**Table:** (b) Isoflurane rule-base [30]

ISO	NB			NM			NS			ZE			PS			PM			PB		
NB																					
																				

NM																					
																				

NS							ZE	PS	ZE	ZE	ZE			ZE					ZE	ZE	
							ZE	ZE	ZE				ZE						PS		

ZE										PS	ZE	PS				ZE	ZE				
							PS	PS	PS	PS	PS	PS				PS			PS		

PS																					
							PS	PS		PS											

PM																					
							PS			PS	PS	PM									

PB																					
							PM	PM		PS											

**Table 4:** Mean and standard deviation of steady state errors and control stabilities of type-1/interval type-2/zSlice general type-2 SOFLC with results of Wilcoxon signed-rank test.

Controller performance	Noise strength	Type	Stage-1 MR	Stage-1 BP	Stage-2 MR	Stage-2 BP
Steady state errors	0.1	1	0.0152 (±) 0.0095	1.3083 (±) 1.3780	0.0622 (±) 0.0229	3.2880 (±) 2.2323
		Interval	0.0057 (±) 0.0013^a^	0.1003 (±) 0.0083^a^	0.0058 (±) 0.0010^a^	0.1160 (±) 0.0085^a^
		General	0.0022 (±) 0.0007^bc^	0.1517 (±) 0.1277^b^	0.0062 (±) 0.0006^b^	0.0309 (±) 0.0206^bc^
	0.2	1	0.0177 (±) 0.0096	2.4974 (±) 1.3428	0.0697 (±) 0.0143	4.1290 (±) 1.6087
		Interval	0.0049 (±) 0.0019^a^	0.1673 (±) 0.0406^a^	0.0036 (±) 0.0014^a^	0.0559 (±) 0.0115^a^
		General	0.0065 (±) 0.0017^b^	0.0253 (±) 0.0189^bc^	0.0098 (±) 0.0011^b^	0.0778 (±) 0.0180^b^

Control stabilities	0.1	1	0.0287 (±) 0.0094	0.6072 (±) 0.3510	0.0159 (±) 0.0005	0.6397 (±) 0.1502
		Interval	0.0096 (±) 0.0009^a^	0.0055 (±) 0.0009^a^	0.0092 (±) 0.0009^a^	0.0052 (±) 0.0006^a^
		General	0.0141 (±) 0.0077^b^	0.0221 (±) 0.0390^b^	0.0110 (±) 0.0012^b^	0.0071 (±) 0.0017^b^
	0.2	1	0.0251 (±) 0.0089	0.8879 (±) 0.3405	0.0194 (±) 0.0036	1.1542 (±) 0.3877
		Interval	0.0145 (±) 0.0019^a^	0.0104 (±) 0.0018^a^	0.0149 (±) 0.0018^a^	0.0115 (±) 0.0026^a^
		General	0.0183 (±) 0.0021^b^	0.0190 (±) 0.0047^b^	0.0170 (±) 0.0013^b^	0.0167 (±) 0.0035^b^

^a^Accept hypothesis: interval type-2 SOFLC is better than type-1 SOFLC.

^b^Accept hypothesis: zSlice general type-2 SOFLC is better than type-1 SOFLC.

^c^Accept hypothesis: zSlice general type-2 SOFLC is better than interval type-2 SOFLC.

**Table 5:** Mean and standard deviation of steady state errors and control stabilities of type-1/interval type-2/zSlice general type-2 SOFLC using extracted rule-base with results of Wilcoxon signed-rank test.

Controller performance	Noise strength	Type	Stage-1 MR	Stage-1 BP	Stage-2 MR	Stage-2 BP
Steady state errors	0.1	1	0.0181 (±) 0.0092^*^	1.6288 (±) 1.5593^*^	0.0515 (±) 0.0296	2.5869 (±) 2.5605^*^
		Interval	0.0069 (±) 0.0012^*^	0.0919 (±) 0.0087	0.0066 (±) 0.0005^*^	0.1292 (±) 0.0045^*^
		General	0.0018 (±) 0.0008	0.1268 (±) 0.0106^*^	0.0056 (±) 0.0007	0.0416 (±) 0.0062^*^
	0.2	1	0.0154 (±) 0.0122	2.1513 (±) 1.4063^*^	0.0730 (±) 0.0032^*^	4.5476 (±) 0.2598^*^
		Interval	0.0052 (±) 0.0022	0.1452 (±) 0.0146	0.0044 (±) 0.0009^*^	0.0839 (±) 0.0100^*^
		General	0.0045 (±) 0.0013	0.0677 (±) 0.0842^*^	0.0079 (±) 0.0012	0.0433 (±) 0.0159

Control stabilities	0.1	1	0.0239 (±) 0.0077	0.6005 (±) 0.3110^*^	0.0147 (±) 0.0014	0.5133 (±) 0.0462
		Interval	0.0083 (±) 0.0007	0.0046 (±) 0.0004	0.0078 (±) 0.0006	0.0043 (±) 0.0003
		General	0.0099 (±) 0.0011	0.0052 (±) 0.0009	0.0099 (±) 0.0005	0.0059 (±) 0.0006
	0.2	1	0.0237 (±) 0.0079^*^	0.9731 (±) 0.5516^*^	0.0171 (±) 0.0009	1.0247 (±) 0.0833
		Interval	0.0134 (±) 0.0012	0.0094 (±) 0.0013	0.0126 (±) 0.0011	0.0083 (±) 0.0011
		General	0.0170 (±) 0.0042	0.0187 (±) 0.0183	0.0148 (±) 0.0011	0.0130 (±) 0.0020

^*^Reject hypothesis: extracted rule-base is better than expert derived rule-base.

**Table:** (a) Atracurium rule-base

Input 1	Linguistic term of input 1	Input 2	Linguistic term of input 2	Output linguistic term	Firing percentage (%)
E_MR	NS	E_BP	NS	PS	1.59
E_MR	NS	IE_BP	NS	PS	1.60
E_MR	NS	E_BP	PS	ZE	1.59
E_MR	NS	IE_BP	PS	ZE	1.60
E_BP	NS	IE_BP	NS	ZE	1.59
E_BP	NS	IE_BP	PS	ZE	1.60
IE_MR	NS	E_BP	PB	ZE	2.92
IE_MR	NS	IE_BP	PB	ZE	2.91
E_MR	ZE	IE_BP	ZE	ZE	6.39
E_BP	ZE	IE_BP	ZE	ZE	5.92
IE_MR	ZE	E_BP	PM	ZE	2.89
IE_MR	ZE	IE_BP	PM	ZE	3.07
E_MR	PS	E_BP	NS	PS	1.60
E_MR	PS	IE_BP	NS	PS	1.60
E_MR	PS	E_BP	PS	ZE	1.60
E_MR	PS	IE_BP	PS	ZE	1.60
E_BP	PS	IE_BP	NS	ZE	1.59
E_BP	PS	IE_BP	PS	ZE	1.60
IE_MR	PS	E_BP	PB	ZE	2.90
IE_MR	PS	IE_BP	PB	ZE	2.91
E_MR	PB	IE_MR	NS	ZE	2.91
E_MR	PB	IE_MR	PS	ZE	2.91
E_MR	NS	E_BP	ZE	PS	3.18
E_MR	ZE	E_BP	ZE	PS	5.92
E_BP	ZE	IE_BP	NS	ZE	3.18
E_BP	ZE	IE_BP	PS	ZE	3.21
IE_MR	ZE	E_BP	PB	ZE	5.41
IE_MR	ZE	IE_BP	PB	ZE	5.78
E_MR	PS	E_BP	ZE	PS	3.21
E_MR	PM	IE_MR	ZE	PS	2.11
E_MR	PB	IE_MR	ZE	PS	5.52

**Table:** (b) Isoflurane rule-base corresponding to each of the decomposed 2-input/1-output systems

Input 1	Linguistic term of input 1	Input 2	Linguistic term of input 2	Output linguistic term	Firing percentage (%)
E_MR	NS	E_BP	NS	PS	1.59
E_MR	NS	IE_BP	NS	ZE	1.60
E_MR	NS	E_BP	PS	ZE	1.59
E_MR	NS	IE_BP	PS	ZE	1.60
E_BP	NS	IE_BP	NS	ZE	1.59
E_BP	NS	IE_BP	PS	PS	1.60
IE_MR	NS	IE_BP	PB	PS	2.91
E_MR	ZE	E_BP	ZE	ZE	5.92
E_MR	PS	E_BP	NS	ZE	1.60
E_MR	PS	IE_BP	NS	PS	1.60
E_MR	PS	E_BP	PS	ZE	1.60
E_MR	PS	IE_BP	PS	ZE	1.60
E_BP	PS	IE_BP	NS	PS	1.59
E_BP	PS	IE_BP	PS	PS	1.60
IE_MR	PS	IE_BP	PB	ZE	2.91
E_MR	PM	IE_MR	ZE	PS	3.07
E_MR	PB	IE_MR	NS	PM	2.91
E_MR	PB	IE_MR	PS	PS	2.91
E_MR	NS	E_BP	ZE	ZE	3.18
IE_MR	NS	E_BP	PB	PS	2.75
E_MR	ZE	IE_BP	ZE	PS	6.39
E_BP	ZE	IE_BP	NS	PS	3.18
E_BP	ZE	IE_BP	ZE	PS	5.92
E_BP	ZE	IE_BP	PS	PS	3.21
IE_MR	ZE	E_BP	PM	PS	2.79
IE_MR	ZE	IE_BP	PM	PS	2.98
IE_MR	ZE	E_BP	PB	PS	5.41
IE_MR	ZE	IE_BP	PB	PS	5.78
E_MR	PS	E_BP	ZE	ZE	3.21
IE_MR	PS	E_BP	PB	PS	2.65
E_MR	PB	IE_MR	ZE	PS	5.78

Note: E_MR: error of muscle relaxation; IE_MR: integration error of muscle relaxation; E_BP: error of BP; IE_BP: integration error of BP.

**Table:** (a) Atracurium rule-base

Input 1	Linguistic term of input 1	Input 2	Linguistic term of input 2	Output linguistic term	Firing percentage (%)
E_MR	NS	E_BP	NS	PS	2.37
E_MR	NS	E_BP	ZE	PS	4.74
E_MR	NS	E_BP	PS	ZE	2.37
E_MR	NS	IE_BP	NS	PS	2.39
E_BP	NS	IE_BP	NS	ZE	2.37
E_MR	ZE	E_BP	ZE	PS	9.19
E_MR	ZE	IE_BP	ZE	ZE	9.56
E_BP	ZE	IE_BP	NS	ZE	4.74
E_BP	ZE	IE_BP	ZE	ZE	9.19
IE_MR	NS	E_BP	PB	PS	4.44
IE_MR	ZE	E_BP	PM	PS	4.41
IE_MR	ZE	E_BP	PB	PS	8.59
IE_MR	PS	E_BP	PB	PS	4.44
E_MR	PM	IE_MR	ZE	PS	4.57
E_MR	PB	IE_MR	ZE	ZE	8.93

**Table:** (b) Isoflurane rule-base corresponding to each of the decomposed 2-input/1-output systems

Input 1	Input linguistic term 1	Input 2	Input linguistic term 2	Output linguistic term	Firing percentage (%)
E_MR	NS	E_BP	NS	PS	2.37
E_MR	NS	IE_BP	NS	ZE	2.39
E_MR	NS	E_BP	ZE	ZE	4.74
E_MR	NS	E_BP	PS	ZE	2.37
IE_MR	NS	E_BP	PB	PS	4.49
E_MR	ZE	E_BP	ZE	ZE	9.19
E_MR	ZE	IE_BP	ZE	PS	9.56
E_BP	ZE	IE_BP	NS	PS	4.74
E_BP	ZE	IE_BP	ZE	PS	9.19
IE_MR	ZE	E_BP	PM	PS	4.46
IE_MR	ZE	E_BP	PB	PS	8.65
IE_MR	PS	E_BP	PB	PS	4.44
E_MR	PB	IE_MR	ZE	PS	8.93
E_MR	PM	IE_MR	ZE	PS	4.50

Note: E_MR: error of muscle relaxation; IE_MR: integration error of muscle relaxation; E_BP: error of BP; IE_BP: integration error of BP.
